# Repetition of anchor stimuli and nature of vocal samples in the perceptual auditory judgment performed by speech-language pathology students

**DOI:** 10.1590/2317-1782/20212021064

**Published:** 2022-01-21

**Authors:** Noemi de Oliveira Bispo, Rosiane Yamasaki, Marina Martins Pereira Padovani, Mara Behlau

**Affiliations:** 1 Centro de Estudos da Voz – CEV - São Paulo (SP), Brasil.; 2 Universidade Federal de São Paulo – UNIFESP - São Paulo (SP), Brasil.; 3 Faculdade de Ciências Médicas Santa Casa de São Paulo - São Paulo (SP), Brasil.

**Keywords:** Voice, Voice Disorders, Dysphonia, Voice Quality, Auditory Perception

## Abstract

**Purpose:**

Verify the effect of anchor repetition in the perceptual auditory judgement of the type of vocal deviation performed by speech-language pathology (SLP) students; analyze the correlation between the amount of different vocal dimensions in the same stimuli and accuracy; investigate the correlation between type of vocal deviation and accuracy.

**Methods:**

54 SLP students were divided in two groups: Group with repetition (GwR), 28 students; and, Group with no repetition (GnR), 26 students. The analyzed sample counted with 220 dysphonic human voices, vowel /ε/. The GwR heard three anchor stimuli before the judgement and every 20 voices during the assessment. The GnR heard the anchor only before beginning the judgement. The anchor stimuli counted with one rough, one breathy and one strain voice. These classifications were compared with reference judgements from three expert SLPs. The intra and inter-rater reliability, the correlation between the amount of different vocal dimensions in the same stimuli and type of vocal deviation with accuracy were assessed.

**Results:**

The accuracy between type of deviation was similar among groups. The GwR presented slightly higher intra and inter-rater reliability. The student’s accuracy was inversely proportional to the amount of different vocal dimensions in the stimuli. Breathiness presented the highest accuracy and strain presented the lowest accuracy.

**Conclusion:**

The repetition of anchor stimuli improved intra and inter-rater reliability. However, it was not effective in the accuracy of the type of vocal deviation. The amount of different vocal dimensions in the stimuli have influence in the students’ accuracy.

## INTRODUCTION

The vocal assessment must be multidimensional, considering the clinician and the patients' point of view. The perceptual judgment of the voice quality (PJVQ) is one of the analysis of the vocal assessment and it is considered to be the gold standard^(1,2)^, widely used for scientific and clinical purposes. The PJVQ enables the characterization of the vocal quality, the vocal degree of deviation, the analysis of the vocal function^(3)^, differential diagnosis in neurological dysphonias^(4)^, follow-up of pre and post-interventions, and the development of clinical analysis for decision making^(5,6)^. In addition, the PJVQ is a simple, accessible, efficient, and low-cost tool^(7,8)^.

The PJVQ is a subjective analysis, hence, it is criticized. Due to its subjectivity, there are more chances of error. Some errors may be random, some are systematic and can be controlled if properly identified. Hence, knowing the source of systematic errors in the PJVQ can provide more control in the assessment reducing its subjectivity and providing a more robust assessment^(1,2,9-12)^. However, the literature still lacks information regarding the source of these systematics errors.

Some factors that contribute to errors in the perceptual judgment of the voice quality are the experience of the judge, the speech task, the type of protocol, the vocal parameters considered in the analysis, the cognitive biases and, the use of anchor stimuli that provides an external reference to the judge. Each judge builds individual mental networks to represent the vocal deviation according to his vocal training and clinical experience; the use of vocal anchors is a strategy that enables the calibration of the auditory perception. In addition, the lack of attention and auditory fatigue can also interfere with the perception of auditory stimuli^(1,2,13,14)^.

The human voice is a complex stimulus and can have many vocal deviations together. Therefore, one single stimulus may present a different type of vocal deviation, such as breathiness and roughness, i.e., two dimensions in one voice^(15)^. Previous studies report that some dimensions are more reliable than others; the most reliable is the overall degree of deviation (G), followed by breathiness (B), and next by roughness (R). The less reliable is strain voice (S)^(1,6,11,16,17)^.

When the judges are exposed to the voices of patients with different vocal deviations, they begin to build internal standards; these standards are also built considering the amount of training, cultural preferences, and personal experiences regarding vocal quality^(2,16,18,19)^. The effect of auditory anchors on the vocal assessment is affected by clinical experience, there is more agreement when the judges have more experience. A judge is considered to be an expert when he has assessed and treated vocal deviations for at least 3 years, this will provide a higher perception regarding the vocal quality^(15)^.

External references, i.e., anchor voices, for normal and deviated vocal quality reduce the internal standard variability that affects the voice judgment^(2)^. The intra and inter-rater reliability are a great challenge for the PJVQ, thus, the use of anchor stimuli seems to be a good strategy to improve the reliability of the judgment and reduce the inter-raters reliability. Both inexperienced and experienced listeners present higher intra and inter-rater reliability when they use an external reference when judging the voice quality, once it reduces the subjectivity of the assessment^(2,16,18)^.

The repetition of anchor stimuli may generate a positive effect in the judge’s perception, with better processing of the stimuli which will influence his decision and his perception when identifying the stimuli. ^(20)^ Thus, the repetition of anchor stimuli during the vocal assessment may help the judge to identify and classify vocal deviation faster and with higher accuracy.

The search of training strategies to teach inexperienced evaluators on the perceptual judgment of the voice quality is highly important; therefore, the aims of the present study were: to verify the effect of anchor repetition in the perceptual auditory judgment of the type of vocal deviation performed by speech-language pathology (SLP) students; analyze the correlation between the number of different vocal dimensions in the same stimuli and its accuracy and, to investigate the correlation between the type of vocal deviation and accuracy. The study hypotheses are: repetition will increase the accuracy of the classification and the intra and inter-rater reliability; the vocal sample characteristics, such as the number of vocal dimensions in each stimulus and type of vocal deviation will influence the accuracy.

## METHODS

### Formation of groups

The study counted with 56 speech-language pathology students. The students were divided into two groups: The Group with No Repetition of anchor stimuli – GnR with 28 students and the Group With Repetition of anchor stimuli – GwR with 28 students. This is a prospective, quasi-experimental study. It was approved by the Committee for Ethics in Research of the *Universidade Cruzeiro do Sul*, under the protocol number 2.994.785; all participants signed an informed consent form. The inclusion criteria were: be a speech-language pathologist student, no previous practice experience in the vocal clinic activities. The exclusion criteria were: the presence of self-reported hearing complaints and incorrectly filled out the protocol used to assess the vocal quality.

### Perceptual auditory judgment of the vocal sample performed by the students

The PJVQ took place in the students' classrooms. The vocal stimuli were presented using a speaker by JBL (model: Flip 5); it was placed in front of the classroom and set at a comfortable loudness in the quiet environment. Before the hearing session began, the students answered if they had any hearing complaints.

The participants were instructed to mark in the protocol the predominant voice deviation of each stimulus. The GwR listened to three anchor stimuli at the beginning of the task and again every 20 voices. The GnR listened to the same anchor stimuli only before beginning the task.

Participants were advised to keep their attention during the entire listening period and to mark the predominant voice deviation in the protocol where: “R” = rough voice, “B” = breathy voice, “S” = strain voice, and “X” = no predominant vocal quality. In addition, the students should mark in the protocol in which voice stimuli they began perceiving auditory fatigue. With no breaks during the hearing session, the PJVQ lasted 1h and 10 minutes for the GwR and, 42 minutes for the GnR.

### Vocal sample

The vocal sample counted with 200 dysphonic human voices previously selected from a voicebank. The speech task was the sustained vowel /ε/ in comfortable pitch and loudness. To assess intra-rater reliability, 20 voices from the sample were randomly chosen and its repetition was added at the end of the analysis. Thus, the analysis had 220 stimuli.

### Anchor stimuli

The anchor stimuli were selected from a voicebank. It counted with three human voices with a moderate degree of vocal deviation to better represent the predominant type of voice deviation. Three types of predominant degrees of deviation were selected: roughness, breathiness, and strain. This selection was performed by three voice specialists with over 10 years of experience in PJVQ.

### Analysis for reference determination

Two analyses to set a reference were developed: 1. To provide the reference for evaluating the students' accuracy regarding the predominant type of voice deviation; 2. to identify the number of vocal dimensions in each stimulus.

In the analysis to provide the reference for the predominant type of voice deviation, three expert judges performed the perceptual auditory judgment of the vocal quality of all 200 voices. This assessment was performed in one hearing session, in a quiet room using loudspeakers. The analysis was defined with the consensus of at least two judges. According to this assessment: 74 voices presented roughness, 67 presented breathiness and, 59 presented strain, as their predominant deviation.

**Figure 1:**
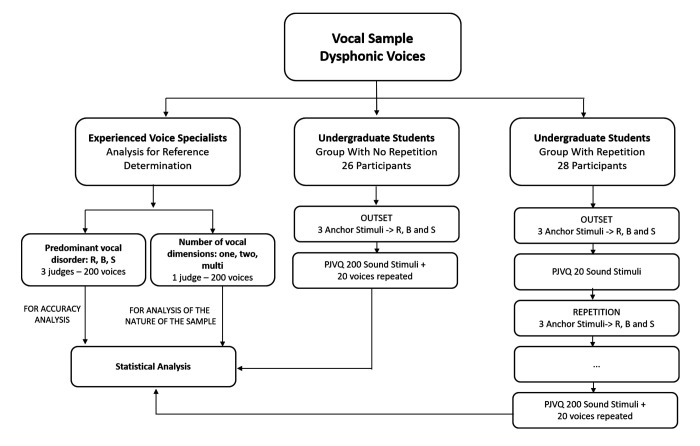
Perceptual judgment of the voice quality diagram performed by the voice specialists and the students

To identify the amount of vocal dimensions in the same stimuli, one expert judge classified the stimuli as one-dimensional, two-dimensional, or multidimensional voices. One-dimensional voices presented one type of vocal deviation, such as roughness, breathiness, or strain. Two-dimensional voices had two predominant deviations, such as roughness and breathiness, roughness and strain, or breathiness and strain. Multidimensional voices presented more than two predominant voice deviations, including instability. The sample did not include voices with instability as the predominant deviation. According to this classification, the sample counted with: 34 one-dimensional voices; 131 two-dimensional voices, and 35 multidimensional voices. Figure 1 shows the PJVQ diagram performed by the voice specialists and the students.

### The predominant type of voice deviation and accuracy

The 200 stimuli were divided into two groups: Accuracy ≥ 75%: vocal stimuli with the occurrence of correct answers above or equal to 75%; Accuracy < 75%: vocal stimuli with the occurrence of correct answers below 75%. The accuracy threshold was set as 75% once it represents a satisfactory accuracy of the answers. Next to this distribution, we determined which vocal dimension – roughness, breathiness, or strain – was more constant in each group.

### Statistical analysis

The Shapiro-Wilk test was used to verify for normal distribution to choose the appropriate statistical tests for comparing the variables (parametric or non-parametric test). The variables were analyzed by both parametric and non-parametric tests, according to the characteristics of the variables.

The following statistical tests were used: Student's t-test for independent samples, Mann-Whitney U test, Spearman correlation for non-parametric tests, Kappa coefficient, Fisher's exact test, Friedman ANOVA, post hoc analysis performed with the Wilcoxon signed-rank test with Bonferroni correction and multiple linear regression.

The significance level was set at 5% (p ≤ 0.05). The SPSS Statistics software, version 25.9 (IBM Corp., Armonk NY, USA) was used. The 95% confidence interval was calculated with the bias-corrected and accelerated approach based on 2000 bootstrap samples.

## RESULTS

Of the 56 students, two were excluded: one did not properly fill out the protocol and, one presented hearing complaints. Thus, the GnR counted with 26 students and the GwR with 28 students, 54 students in total. The predominant type of voice deviation was the analyzed parameter.

### Accuracy of the predominant type of vocal deviation

The comparison between the student's analysis and the reference analysis (established by three expert voice specialists) showed similar outcomes for the GnR and the GwR. The average accuracy percentage for the GnR and GwR was 54.65% and 52.29%, with a standard deviation of 6.04 and 5.47, respectively. The minimum and maximum percentage of correct answers for the GnR were 43.67% and 65%. The GwR minimum correct answers were 38.18% and the maximum 63.64%. In this analysis, the Effect Size was 0.391^d^, p-value = 0.137 ^a^. The analysis of these data considered the Student's t-test (parametric) for independent samples and the Mann-Whitney U test (non-parametric). The effect size between groups was measured with coefficient r or d.

### Intra-rater reliability

To assess the intra-rater reliability, 10% of the sample was repeated and the Kappa coefficient was used. The GwR presented higher intra-rater reliability than the GnR, an average of 0.431 (minimal reliability) and 0.359 (weak reliability), respectively, p-value < 0.050.

### Inter-rater reliability

The inter-rater reliability was assessed using the Kappa coefficient. The GwR presented higher inter-rater reliability when compared to the GnR, an average of 0.276 and 0.247, respectively, p-value < 0.001. Both groups presented minimal reliability.

### Correlation of the number of different vocal dimensions and its accuracy

Table 1 presents that the students' accuracy was different regarding the number of different vocal dimensions in the same vocal stimuli, for both groups and in total, p-value <0.001. The sample had 17% one-dimensional voices; 65.5% two-dimensional voices and 17.5% multidimensional voices.

**Table 1:** Descriptive values and comparative analysis for identifying the predominant type of vocal deviation, according to the number of vocal dimensions (1 - one-dimensional, 2 - two-dimensional, 3 - multidimensional) for the GnR, GwR, and total sample

**Variable**	**Groups**	**Number of vocal dimensions**	**Average**	**SD**	**Median**	**Min.**	**Max.**	**p-value**	**Post-hoc**	**p-value**	r
**Accuracy (%)**	**GnR**	1	72.29	14.65	76.47	26.47	91.18	**< 0.001^a^** ^*^	1D x 2D	**0.001**	0.577
		2	50.70	6.46	49.62	37.40	64.89		1D x 3D	**0.001**	0.635
		3	48.46	9.61	51.43	31.43	65.71		2Dx 3D	0.999	0.058
	**GwR**	1	67.65	10.12	67.65	52.94	85.29	< 0.001^a^*	1D x 2D	**0.001**	0.500
		2	49.54	6.25	50.00	35.88	61.83		1D x 3D	**0.001**	0.946
		3	41.94	7.93	42.86	28.57	54.29		2Dx 3D	**0.003**	0.446
	**Total**	1	69.88	12.61	73.53	26.47	91.18	< 0.001^b^*	1D x 2D	**0.001**	1.569
		2	50.10	6.32	49.62	35.88	64.89		1D x 3D	**0.001**	1.967
		3	45.0	9.30	45.71	28.57	65.71		2Dx 3D	**0.001**	0.795

**Caption:** D: dimensions; SD: Standard deviation; Min.: Minimum; Max.: Maximum

Friedman ANOVA (^a^) and ANOVA with repeated measures (^b^)

Test for effect size: r coefficient (r)

* statistically significant value at 5% (p ≤ 0.05)

The GwR presented a higher percentage of correct answers when the vocal alteration was considered one-dimension compared to two-dimensions or multidimensions. Also, the percentage of correct answers was higher when the vocal deviation considered two dimensions compared to three dimensions. This investigation used the post hoc analysis with the Wilcoxon signed-rank test with Bonferroni correction.

The GnR also presented a higher percentage of correct answers when the vocal alteration was considered one-dimension compared to two-dimensions or multidimensions. The percentage of correct answers for two-dimensional and three-dimensional voices was similar.

**Figure 2:**
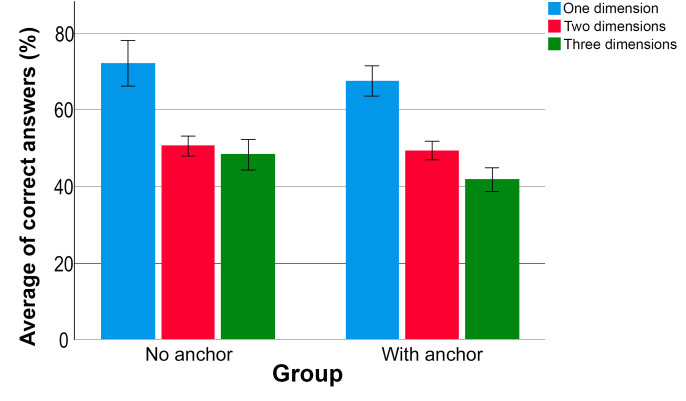
Average of correct answers per group according to the number of altered voice dimensions in the stimuli

Considering both groups, i.e., the total sample, the post hoc analysis test with Bonferroni correction showed that voices with vocal deviation in one dimension present a higher percentage of correct answers than voices with vocal deviations in two or more dimensions. In addition, more correct answers were observed for voices with deviation in two dimensions than in multidimensions. Thus, voices with deviation in more dimensions, i.e., roughness, breathiness, strain, and instability, reduce the listeners' accuracy in the assessment. Figure 2 presents the average of correct answers per group according to the number of altered voice dimensions in the same stimuli.

### Number of vocal dimensions: multiple linear regression

Multiple linear regression models were designed to verify the predictive capacity of the number of vocal dimensions concerning the performance of identifying the predominant vocal deviation, for each group and the total sample. In these models, the percentage of correct answers for each vocal stimulus was considered as a dependent variable and the number of altered dimensions as an independent variable. The vocal dimension - roughness, breathiness, or strain – was initially added as the first step in the models, to control its effect. The independent variable, i.e., the number of altered vocal dimensions, was inserted in the second step.

The results improved significantly in the linear regression model concerning the predictive capacity of the performance of identifying the predominant vocal deviation after adding the number of altered dimensions, indirect order. The variance in the number of altered dimensions explains 12%, 16.1%, and 16% of the observed variance in the GnR, GwR, and total sample, respectively, to identify the predominant vocal deviation. The variable number of vocal dimensions contributed significantly to the models, p < 0.001, and the non-standardized coefficient b suggested that, when controlling the other variables in the model, the increase in an altered dimension leads to a reduction of 13.13% of correct answers in the GnR, 15.51% of correct answers in the GwR and 14.36% of correct answers in the total sample. Thus, the number of vocal dimensions was a significant predictor in the identification of the predominant vocal deviation, for both groups and the total sample, Table 2.

**Table 2:** Multiple linear regression model of the number of altered dimensions as a predictor of the performance of identifying the predominant vocal deviation, for GnR, GwR, and total sample

**Step**		**b**	**β**	**p-value**
**Group With No Anchor Repetition – GnR**				
1	Constant	58.28	--	**< 0.001** ^*^
	Predominant type of voice deviation	-2.23	-0.083	0.240
		-5.81; 1.34		
2	Constant	88.61	--	< 0.001*
	Predominant type of voice deviation	-4.31	-0.161	0.019*
		-7.49; -0.96		
	Number of altered dimensions	-13.13	-0.355	< 0.001*
		-18.11; -8.03		
**Group With Anchor Repetition – GwR**				
1	Constant	63.72	--	< 0.001*
		56.04; 71.18		
	Predominant type of voice deviation	-6.46	-0.209	0.003*
		-10.75; -1.91		
2	Constant	99.56	--	< 0.001*
		85.52; 113.32		
	Predominant type of voice deviation	-8.92	-0.289	< 0.001*
		-12.54; -4.72		
	Number of altered dimensions	-15.51	-0.363	< 0.001*
		-21.33; -9.73		
**Total**				
1	Constant	61.10	--	< 0.001*
	Predominant type of voice deviation	-4.42	-0.164	0.020*
		-8.32; -0.67		
2	Constant	94.28	--	< 0.001*
		81.88; 105.73		
	Predominant type of voice deviation	-6.70	-0.249	< 0.001*
		-10.03; -3.34		
	Number of altered dimensions	-14.36	-0.386	< 0.001*
		-19.14; -9.66		

r^2^ = 0.007 (p = 0.240) for Step 1; r^2^ = 0.120 (p < 0.001*) for Step 2 for GnR; r^2^ = 0.044 (p = 0.003*) for Step 1; r^2^ = 0.161 (p < 0.001*) for Step 2 for GwR; r^2^ = 0.027 (p = 0.020*) for Step 1; r^2^ = 0.160 (p < 0.001*) for Step 2 for the total

* statistically significant value at 5% (p ≤ 0.05).

### Correlation between the predominant type of voice deviation and accuracy

The comparison of the GwR and the GnR with the reference showed differences among groups when analyzing strain. The GnR had higher accuracy when classifying vocal strain than GwR, p<0.001. The other vocal deviation had similar outcomes, Table 3.

**Table 3:** Descriptive values and comparison of groups regarding the percentages of correct answers when identifying the predominant type of vocal deviation and the responses when no predominant vocal deviation was identifie

**ACCURACY**	**Group**	**Average**	**SD**	**Median**	**Min.**	**Max.**	**p-value**	**E.S**
**Roughness**	GnR	55.86	9.32	54.88	41.46	71.95	0.992^a^	0.003^d^
	GwR	55.84	10.27	56.10	26.83	73.17		
**Breathiness**	GnR	57.13	10.04	59.34	32.00	70.67	0.556^a^	0.148^d^
	GwR	58.62	8.40	58.00	45.33	73.33		
**Strain**	GnR	50.12	9.80	50.79	30.16	66.67	**< 0.001** ^*^ **^a^**	1.019^d^
	GwR	40.14	9.04	39.68	25.40	55.56		
**No predominant deviation**	GnR	2.15	3.28	0.45	0.00	10.00	0.121^b^	0.220^r^
	GwR	3.07	3.64	1.82	0.00	13.18		

**Caption:** SD: Standard deviation; Min.: Minimum; Max.: Maximum; E.S.: Effect Size

T-Student test for independent samples (^a^) and Mann-Whitney U test (^b^)

* statistically significant value p ≤ 0.05

Test for effect size: coefficient r (^r^) and coefficient d (^d^)

The post hoc analysis, performed with the Bonferroni test, for all assessed voices, revealed that the repetition of anchor stimuli was not related with higher or lower accuracy, with no group effect, p = 0.072, r = 0.247. However, considering the predominant vocal deviation, there was a significant effect, p < 0.001, r = 0.600. Strain presented lower accuracy: “strain” and “roughness”, p < 0.001; “strain” and “breathiness”, p < 0.001. No difference was observed between “roughness” and “breathiness”, p = 0.743. Therefore, considering both groups, strain voices have lower accuracy when compared to rough and breathy voices. The accuracy of the analysis for the type of vocal dimension was different for each group, with statistically significant interaction, p = 0.003, r = 0.323.

To verify high or low accuracy according to the predominant type of vocal deviation, breathiness, roughness, or strain, the stimuli were divided into two groups: the number of correct answers ≥ to 75% and < 75%. The relation of the number of correct answers and all the stimuli showed that the proportions of breathy, rough, and strain voices were similar in the groups with correct answers above or equal to 75% and smaller than 75%, both for GnR and GwR. However, considering the total sample, breathiness presented more frequently accuracy ≥ 75%, Table 4.

**Table 4:** Comparison of the type of vocal deviation regarding the accuracy for each group and total sample

**GROUP**	**Predominant Vocal Deviation**	**Accuracy**			**p-value**
		**High**	**Low**	**Total**	
		Number (%)	Number (%)	Number (%)	
**GnR**	R	12 (28.57)	62 (39.24)	74 (37.00)	0.311
	B	18 (42.86)	49 (31.01)	67 (33.50)	
	S	12 (28.57)	47 (29.75)	59 (29.50)	
**GwR**	R	11 (25.58)	63 (40.13)	74 (37.00)	0.053
	B	21 (48.84)	46 (29.30)	67 (33.50)	
	S	11 (25.58)	48 (30.57)	59 (29.50)	
**Total**	R	9 (25.71)^a^	65 (39.39)^a^	74 (37.00)	**0.023** ^*^
	B	19 (54.29)^b^	48 (29.09)^a^	67 (33.50)	
	S	7 (20.00)^a^	52 (31.52)^a^	59 (29.50)	

The letters (a,b) indicate subsets of the variable “Group” whose proportions of the columns do not differ significantly from each other at the 5% significance level (p ≤ 0.05).

* statistically significant value p ≤ 0.05

### Accuracy regarding the self-reported auditory fatigue

Univariate analysis of variance (ANOVA) was performed to compare the effect of self-reported fatigue on the accuracy of their responses. This analysis, considered only 23 participants of the GnR and 21 participants of the GwR, since some participants did not report fatigue. The mean accuracy percentage pre-fatigue for the GnR was 55.77% and for the GwR it was 55.18%. In the post-fatigue moment, the GnR had an average percentage of 53.91% while the GwR had 52.65%. The effect size of the factors was measured using the r coefficient, calculated considering the conversion of the F statistic as proposed by Field. No statistically significant interactions were observed between the factors “Group” and “Fatigue Moment” for the percentage of correct answers, p = 0.788.

Thus, the difference between the participants in the groups with and with no anchors repetition, regarding the accuracy of the voice task, was not influenced by self-reported fatigue. Hence, the pre- and post-fatigue moments were similar.

## DISCUSSION

The perceptual judgment of the voice quality is of great clinical value; therefore, it is widely used in clinical practice^(5)^. Nonetheless, it is a subjective analysis that may present random and systematic errors^(12)^. The systematic errors are easier to be controlled, however, their source must be known. The judge’s training influences the PJVQ. Inexperienced judges need at least 8 hours of training to obtain acceptable reliability^(21)^. The perceptual training, with human or synthesized voices, creates internal standards of the vocal stimuli. The use of anchor stimuli also influences the PJVQ once it enables the calibration of the auditory perception.

The PJVQ also suffers from the influence of the vocal task. The present study considered the sustained vowel /ε/, in comfortable pitch and loudness, for the analysis. The sustained vowel is commonly used in clinical practice and research once it provides information regarding the vocal source, with no interference of the articulation and prosody^(7)^. Vocal deviations such as roughness and breathiness are produced by the larynx; the larynx works as a transducer of aerodynamic energy, provided from the lungs, into acoustic energy^(22)^. Vocal strain can be produced by the larynx or the vocal tract. Therefore, using sustained vowels in auditory training is an important task to identify the predominant vocal deviation.

The internal standards of inexperienced judges are mostly stable to analyze normal voices since these judges are familiar and used to hearing these types of stimuli, which has previously created their internal standard for perceptual judgment^(2)^. To efficiently prepare the perceptual auditory training, the standard auditory perception of the students must be known. Participants from both groups presented similar outcomes in the accuracy when judging the predominant vocal deviation; 54.65% for the GnR and, 52.29% for the GwR. The low accuracy was expected once the participants were students with little experience in judging dysphonic voices. A previous study with voice specialists’ students observed an average value of accuracy equal to 72%^(23)^.

The repetition of the anchor stimuli during the perceptual judgment did not increase the accuracy of the students from the GwR, contrary to the initial hypothesis of the study. The stimuli repetition helps the brain to recognize standards faster and contributes to the refinement of the stimuli characteristics^(20)^. The accuracy did not increase, however, the repetition strategy increased the intra and inter-rater reliability in the GwR, even though it still presented low values. These difference among groups suggests that the GwR could adjust its internal standards^(16,24,25)^.

The reliability reflects the ability to judge a vocal stimulus in the same way in two different moments. The reliability is influenced by many factors, hence, it is a great challenge, even for experienced judges^(6,18)^. As presented in this study, using reference stimuli, i.e., anchors, might contribute to higher inter-rater reliability. According to the literature^(16,24,26,27)^, the perceptual auditory training with the use of anchor stimuli increases the reliability of inexperienced judges when classifying vocal deviations. Also, inexperienced judges may present the same intra and inter-rater reliability as experienced judges post-training. Therefore, using anchor stimuli potentially reduces the variability when classifying voices and increases the intra and inter-rater reliability of inexperienced listeners as initially hypothesized. The higher reliability indicates that the participants were able to adapt to the external references and adjust their internal standards^(25)^. Although the GwR presented higher reliability compared to the GnR, the reliability value was minimal. Hence, constant perceptual auditory training is needed throughout the students' graduate program.

Regarding the inter-rater reliability, inexperienced judges presented improvement in the reliability after two hours of training using anchor stimuli with different types of vocal deviation^(16)^. The use of anchor stimuli when assessing and classifying vocal deviations, reduce and might even eliminate several internal standards, reducing interferences and increasing the inexperienced and experienced raters’ reliability^(18,24)^. In the present study, the inter-rater reliability was minimal for both groups, it was lower than the intra-rater reliability; however, the values were significantly higher in the GwR. Thus, the use of vocal anchors can be a positive resource that enables the calibration of the auditory perception in students when assessing vocal deviations.

Dysphonic human voices can contain one type of vocal deviation, such as breathiness in cases of vocal fold paralysis; or it can be a complex signal, with two or more altered vocal dimensions. The assessment of these complex vocal stimuli requires a more refined auditory perception, once it is harder to identify the predominant vocal deviation^(1,16)^. As presented in the present study, the presence of more vocal dimensions reduces the listeners' accuracy in the assessment, as seen in Table 1 and Figure 2; this is in accordance with the study hypothesis.

Different factors interfere in the classification of dysphonic voices^(12)^, and the increase of the vocal dimensions in the vocal stimuli was a significant predictor in the classification of the predominant vocal deviation. Although the values were 16.1% for the GwR and 12% for the GnR, the number of different vocal dimensions in the same stimuli was responsible for some of the systematic errors in the PJVQ, Table 2. Hence, the number of vocal dimensions in the same stimuli must be considered in the planning of the auditory training of inexperienced judges; the training must begin with simple stimuli and next include complex stimuli^(1)^.

The GwR and GnR presented similar accuracy when classifying breathy and rough voices. However, the GnR presented a higher percentage of correct answers for strain voices when compared to the GwR (Table 3); even though no anchor repetition was presented throughout the analysis. Therefore, anchor repetition did not improve the internal standard of strain, since the GwR did not present better outcomes compared to the GnR for this parameter^(6,11,16)^. The literature states that strain is more difficult to be analyzed and has lower reliability^(1,12,17)^.

Listeners may be more sensitive to specific vocal qualities because the stimulus is easier to be recognized and identified. The breathy voice presented higher accuracy, Table 4. Considering the total sample analysis, breathy voices had higher accuracy while strain voices presented lower accuracy, as expected. Rough and strain voices had a low rate of correct answers (≥ 75%), differently from the high rate observed in breathy voices. Indeed, vocal deviation may be unstable and include more than one qualitative parameter^(16)^; also, some vocal dimensions are easier to be perceived and memorized.

The rater's experience in classifying voices impacts the PJVQ. Inexperienced raters are not familiar with deviated vocal qualities; thus, they do not have internal standards regarding types of vocal deviations. More experience will allow the rater to place the internal standards consistently throughout the assessment of vocal deviations and to differentiate the vocal dimensions^(16)^.

The present study counted with large sample size, 220 voices. The GwR took more time to complete the PJVQ than the GnR. Fatigue can reduce cognitive performance after long periods of attention-demanding activity^(28-30)^; however, in the present study, the self-reported fatigue did not influence directly the accuracy values, neither in the beginning nor at the end of the assessment.

Perceptual auditory training to improve the student’s perception since the beginning of the undergraduate program may contribute to increasing the reliability and accuracy in their assessment^(16,24)^. Considering the present study outcomes, the perceptual auditory training of students should use anchor voices and begin with simple stimuli, i.e., one-dimension, and next use more complex stimuli. Also, the training must begin with more reliable dimensions, breathiness, roughness, and then proceed to less reliable dimensions, such as strain.

Voices with deviation frequently present more than one type of vocal deviation in the PJVQ, hence, the assessment by inexperienced raters is harder. The anchor stimuli are a useful clinical tool for training listeners with low intra and inter-rater reliability to classify voices^(15,16)^. However, the vocal sample characteristics must be considered when performing perceptual auditory training, contemplating the complexity of the stimuli and the types of vocal deviation.

## CONCLUSION

The repetition of anchor stimuli did not increase the accuracy to classify the predominant type of vocal deviation. The intra and inter-rater reliability increased in the GwR, however, it was still weak. Complex vocal stimuli influenced the accuracy outcome. Simple stimuli had higher accuracy to identify the predominant type of vocal deviation. Therefore, the number of vocal dimensions was a predictor in the classification of the vocal deviation. The type of vocal deviation influenced the accuracy; breathy voices presented more correct answers.
