# Supplementary material for: Repetition of anchor stimuli and nature of vocal samples in the perceptual auditory judgment performed by speech-language pathology students
Source: Codas. 2022 Jan 21;34(4):e20210064. doi: 10.1590/2317-1782/20212021064 (PMC9886121; doi:10.1590/2317-1782/20212021064)
Supplement: Translation

# Translation: Repetição de estímulos âncoras e natureza das amostras vocais no julgamento perceptivo-auditivo realizado por estudantes de fonoaudiologia

**Authors:** Noemi de Oliveira Bispo, Rosiane Yamasaki, Marina Martins Pereira Padovani, Mara Behlau

**Affiliations:** 1 Centro de Estudos da Voz – CEV - São Paulo (SP), Brasil.; 2 Universidade Federal de São Paulo – UNIFESP - São Paulo (SP), Brasil.; 3 Faculdade de Ciências Médicas Santa Casa de São Paulo - São Paulo (SP), Brasil.

**Keywords:** Voz, Distúrbios da voz, Disfonia, Qualidade da Voz, Percepção Auditiva

## Abstract

**Objetivo:**

Verificar o efeito da repetição de estímulos âncoras no julgamento perceptivo auditivo do desvio vocal predominante realizado por estudantes de fonoaudiologia; analisar a relação entre o número de dimensões vocais dos estímulos sonoros e a acurácia; e investigar a relação entre o desvio vocal predominante e a acurácia.

**Método:**

Participaram da pesquisa 54 estudantes de fonoaudiologia, divididos em: Grupo Com Repetição - GCR com 28 alunos; e Grupo Sem Repetição - GSR, 26 alunos. A amostra vocal consistiu de 220 vozes humanas disfônicas, vogal /ε/. O GCR escutou três estímulos âncoras, uma voz rugosa, uma soprosa e uma tensa, no início da tarefa e a cada 20 vozes. O GSR escutou os estímulos âncoras somente no início da tarefa de avaliação. Estas classificações foram comparadas com respostas referências produzidas por 3 fonoaudiólogas experientes. Analisamos a acurácia e a confiabilidade intra e interavaliadores, a correlação entre número de dimensões vocais e acurácia e a relação entre desvio vocal predominante e acurácia.

**Resultados:**

Os grupos tiveram desempenhos semelhantes na acurácia do desvio vocal predominante. A confiabilidade intra e interavaliadores foi discretamente maior no GCR. A acurácia dos alunos foi inversamente proporcional ao número de dimensões vocais presentes nos estímulos. O desvio vocal de maior acurácia foi a soprosidade, e a de menor, a tensão.

**Conclusão:**

A repetição dos estímulos âncoras melhorou a confiabilidade dos estudantes, mas não se mostrou efetiva na acurácia da classificação do desvio vocal predominante. O número de dimensões vocais nos estímulos sonoros interferiu na acurácia dos alunos.

## INTRODUÇÃO

A avaliação vocal deve ser realizada de forma multidimensional, considerando tanto a perspectiva do clínico como a do próprio paciente. Dentre os instrumentos da avaliação vocal, a análise perceptivo-auditiva da voz (APA) é considerada o padrão-ouro^(1,2)^, sendo amplamente utilizada para fins científicos e clínicos. A APA possibilita a descrição da qualidade e da magnitude do desvio vocal, a análise da funcionalidade vocal^(3)^, a realização do diagnóstico diferencial das disfonias neurológicas^(4)^, o acompanhamento dos resultados pré e pós-intervenção e o desenvolvimento do raciocínio clínico para tomada de decisão^(5,6)^. Além disso, a APA é um instrumento simples, acessível, de baixo custo e eficiente^(7,8)^.

Uma das maiores críticas à APA está relacionada com a subjetividade envolvida nessa forma de avaliação. Por ser subjetiva, pode ser mais suscetível a erros. Embora uma fonte de erros na APA possa ser aleatória, outras podem ser sistemáticas e, quando identificadas, tornam-se mais passíveis de controle. Assim, o conhecimento de fontes de erros sistemáticos na APA permite maior controle da avaliação, diminui a subjetividade envolvida e contribui para uma avaliação mais robusta^(1,2,9-12)^. Porém, ainda carecemos de informações da literatura sobre as fontes de erros sistemáticos na APA.

Os fatores que interferem na avaliação perceptivo-auditiva já descritos pela literatura estão relacionados com: experiência do avaliador, tarefas de fala, protocolo de avaliação, escolha dos parâmetros vocais, vieses cognitivos e uso de estímulos âncoras, por meio do qual cria-se uma referência externa para os avaliadores. Como cada avaliador desenvolve representações mentais dos desvios vocais de acordo com o treinamento e a experiência clínica, o uso de estímulos âncoras é uma estratégia que possibilita a calibração da percepção auditiva dos avaliadores. Além desses fatores, a falta de atenção e a fadiga auditiva também podem interferir na percepção dos estímulos sonoros^(1,2,13,14)^.

A voz humana é um estímulo complexo e pode ser composta por um conjunto de desvios vocais. Dessa forma, um único estímulo sonoro pode conter diferentes tipos de vozes, como soprosidade e rugosidade, ou seja, duas dimensões numa única voz^(15)^. Estudos anteriores relatam que algumas dimensões vocais produzem maior confiabilidade do que outras. Em ordem de maior concordância interavaliadores, temos o grau geral do desvio vocal (G), a soprosidade (S) e a rugosidade (R); e o de menor concordância a tensão (T)^(1,6,11,16,17)^.

A exposição dos avaliadores a vozes de pacientes com distúrbios vocais ajuda a moldar os padrões individuais de escuta, assim como o tempo de treinamento, as preferências culturais e os conceitos pessoais sobre a qualidade vocal^(2,16,18,19)^. O grau da representatividade do padrão interno é afetado pela experiência clínica, sendo que a concordância perceptivo-auditiva se mostra maior em indivíduos experientes. Um avaliador é considerado experiente quando avaliou e tratou distúrbios vocais por pelo menos 3 anos, o que resulta no aumento da percepção do profissional em relação à qualidade vocal^(15)^.

Os padrões internos que podem causar variação na classificação das vozes, exercem menor influência quando o avaliador tem acesso a referências externas de vozes normais e desviadas^(2)^. Como a confiabilidade intra e interavaliadores representa um grande desafio para a APA, o uso de estímulos âncoras parece ser uma boa estratégia para aumentar a consistência da avaliação e diminuir a variação de julgamento interavaliadores. Tanto avaliadores experientes como inexperientes apresentam maior concordância intra e interavaliadores quando utilizam referências externas para avaliação perceptiva da voz, pois reduz a subjetividade da avaliação^(2,16,18)^.

A repetição de âncoras pode causar efeito positivo na percepção do avaliador, como um melhor processamento do estímulo repetido, influenciando o tempo de decisão e a precisão no reconhecimento do estímulo^(20)^. Assim, repetir estímulos âncoras ao longo da tarefa de avaliação de vozes, pode contribuir para que os ouvintes reconheçam os tipos de desvios vocais com maior agilidade e acurácia.

Considerando a importância de se buscar estratégias para a realização do treinamento perceptivo-auditivo de avaliadores pouco experientes, esta pesquisa teve como objetivos verificar o efeito da repetição de estímulos âncoras na análise perceptivo-auditiva do desvio vocal predominante realizada por estudantes de fonoaudiologia; analisar a relação entre o número de dimensões vocais dos estímulos sonoros e a acurácia; e investigar a relação entre o desvio vocal predominante e a acurácia. Como hipóteses, a repetição de estímulos âncoras aumenta a acurácia das classificações e a confiabilidade intra e interavaliadores; a natureza das amostras vocais, como o número de dimensões vocais nos estímulos sonoros e o tipo de desvio vocal predominante influenciam a acurácia das classificações.

## MÉTODO

### Formação dos grupos

Participaram deste estudo 56 estudantes de graduação do curso de fonoaudiologia. Os estudantes foram alocados em dois grupos: Grupo Sem Repetição de estímulos âncoras – GSR composto por 28 alunos e Grupo Com Repetição – GCR composto por 28 alunos. Estudo prospectivo, quasi-experimental, aprovado pelo Comitê de Ética em Pesquisa da Universidade Cruzeiro do Sul, sob o parecer nº 2.994.785 e todos os participantes assinaram o Termo de Consentimento Livre e Esclarecido. Os critérios de inclusão foram: ser estudante de fonoaudiologia e ainda não ter participado de atividades práticas no ambulatório de voz clínica. Os critérios de exclusão foram: presença de queixa auditiva autorreferida, não ter preenchido adequadamente o protocolo de análise perceptivo-auditiva.

### Análise perceptivo-auditiva das vozes realizada pelos alunos

A APA foi realizada na sala de aula dos estudantes. Os estímulos sonoros foram apresentados com a utilização de uma caixa de som, marca JBL, modelo *Flip* 5 portátil, posicionada na frente da sala de aula, em intensidade confortável e em ambiente silencioso. Antes de iniciar a sessão de escuta, os alunos assinalaram, no protocolo impresso, se tinham queixa auditiva.

Os participantes foram orientados a marcar no protocolo o desvio vocal predominante de cada um dos estímulos sonoros apresentados. O GCR escutou os três estímulos âncoras no início da tarefa e a cada 20 vozes. O GSR escutou os mesmos estímulos âncoras apenas no início da tarefa.

Os participantes foram instruídos a manter a atenção durante o período de escuta e a marcar no protocolo de avaliação o desvio vocal predominante, sendo “R” para voz rugosa, “S” para voz soprosa, “T” para voz tensa e “X” quando os participantes não detectavam predomínio vocal. Além disso, os alunos foram orientados a marcar no protocolo de avaliação o número da voz em que começaram a perceber fadiga auditiva. Sem intervalos, a duração da APA do GCR e do GSR foi de 1h e 10 minutos e de 42 minutos, respectivamente.

### Amostra vocal

A amostra vocal foi composta por 200 vozes humanas disfônicas selecionadas de um banco de vozes. A tarefa de fala utilizada para a análise foi a emissão da vogal sustentada /ε/ em frequência e intensidade confortáveis, autosselecionadas. Para análise da confiabilidade intra-avaliadores, foram adicionadas 20 vozes repetidas ao fim da amostra, escolhidas aleatoriamente. Sendo assim, a amostra total foi composta por 220 estímulos sonoros.

### Estímulos âncoras

Os estímulos âncoras selecionados foram extraídos de um banco de vozes e consistiram de três vozes humanas desviadas de grau moderado para melhor evidenciar o predomínio vocal. Foram selecionadas uma voz predominantemente rugosa, uma soprosa e uma tensa. Três avaliadoras especialistas em voz com mais de 10 anos de experiência em APA selecionaram os estímulos âncoras.

### Análises de referência

Duas análises de referência foram desenvolvidas para esse estudo: uma para servir de base para avaliação da acurácia dos alunos quanto à classificação do predomínio do desvio vocal; e outra para identificação do número de dimensões vocais nos estímulos sonoros.

Para a análise de referência do desvio vocal predominante, três avaliadoras experientes realizaram a análise perceptivo-auditiva das 200 vozes, numa mesma sessão de escuta, em ambiente silencioso e com a utilização de caixa acústica externa para amplificação do som. A classificação das vozes foi realizada a partir do consenso de pelo menos duas avaliadoras. A partir dessa classificação, 74 vozes foram consideradas predominantemente rugosas, 67 soprosas e 59 tensas.

Para a análise do número de dimensões vocais, uma avaliadora experiente classificou os estímulos sonoros em vozes unidimensionais, bidimensionais ou multidimensionais. As vozes unidimensionais tinham como característica a presença de um desvio vocal, podendo ser rugosa, soprosa ou tensa. As vozes bidimensionais continham a associação de dois desvios, como rugosidade e soprosidade, rugosidade e tensão ou soprosidade e tensão. As vozes multidimensionais podiam conter a associação de mais de dois desvios vocais, inclusive de instabilidade. A amostra vocal não contém vozes com predomínio de instabilidade. A partir dessa classificação, 34 vozes foram avaliadas como unidimensionais, 131 como bidimensionais e 35 como multidimensionais. A Figura 1 mostra um diagrama da APA realizada pelas fonoaudiólogas experientes e pelos alunos de graduação.

**Figura 1:** Diagrama das análises perceptivo-auditivas realizadas por fonoaudiólogos experientes e por alunos de graduação

### Relação entre o desvio vocal predominante e a acurácia

Os 200 estímulos sonoros foram distribuídos em dois grupos: Acurácia ≥ 75%: estímulos sonoros que tiveram ocorrência de acertos entre os participantes maior ou igual a 75%; Acurácia < 75%: estímulos sonoros que tiveram ocorrência de acertos entre os participantes menor que 75%. Optamos por utilizar o limite de separação de 75% por representar uma porcentagem de acurácia satisfatória. Sendo assim, após esta distribuição, apuramos qual dimensão vocal – soprosa, tensa ou rugosa – foi mais constante em cada um dos grupos.

### Análise estatística

Os dados foram analisados estatisticamente, sendo utilizado o teste Shapiro-wilk para verificação do cumprimento do pressuposto de normalidade, de modo a auxiliar na decisão da escolha do teste para comparação das variáveis (teste paramétrico ou não paramétrico). As variáveis foram então analisadas tanto por testes paramétricos quanto por não paramétricos, de acordo com as características das variáveis.

Para a análise das variáveis deste estudo, foram utilizados os seguintes testes: t de Student para as amostras independentes, U de Mann – Whitney, Correlação de Spearman para testes não paramétricos, coeficiente Kappa, Exato de Fisher, ANOVA de Friedman, análise *post hoc* realizada com o teste de postos sinalizados de Wilcoxon com correção de Bonferroni e regressão linear múltipla.

O valor de significância estatística adotado foi igual a 5% (p ≤ 0,05). Utilizou-se o software *SPSS Statistics*, versão 25.9 (IBM Corp., Armonk NY, EUA). Para o cálculo dos intervalos de confiança de 95%, foi utilizado o método de viés corrigido e acelerado com base em 2000 amostra *bootstrap*.

## RESULTADOS

Dos 56 alunos, 2 foram excluídos da pesquisa: um pela marcação incompleta no protocolo de avaliação e o outro, por referir queixa auditiva. Dessa forma, o GSR foi composto por 26 alunos e o GCR por 28, totalizando 54 indivíduos. O parâmetro de análise foi o desvio vocal predominante.

### Acurácia na análise do desvio vocal predominante

A comparação da análise do desvio vocal predominante dos estudantes com a da análise de referência, realizada pelas três avaliadoras experientes, mostrou que o GSR e o GCR tiveram desempenhos semelhantes, com porcentagem média de acurácia de 54,65% e 52,29% e desvio padrão de 6,04 e 5,47, respectivamente. O GSR teve como valor mínimo de acertos 43,67% dos estímulos sonoros e o máximo de acertos de 65%. O GCR teve como valor mínimo de acertos 38,18% dos estímulos sonoros e o máximo de 63,64%. Para esta análise, o Tamanho de Efeito foi de 0,391^d^ e o valor de p = 0,137 ^a^. Para comparar os grupos em relação a estas variáveis, foram utilizados os testes t de Student (paramétrico) para amostras independentes e teste U de Mann-Whitney (não-paramétrico). O tamanho do efeito da diferença entre os grupos foi medido por meio do cálculo do coeficiente r ou d.

### Confiabilidade intra-avaliadores

A concordância intra-avaliadores foi verificada por meio da repetição de 10% da amostra e da aplicação do coeficiente Kappa. O GCR apresentou confiabilidade intra-avaliadores significantemente maior que o GSR, média de 0,431 (concordância mínima) e 0,359 (concordância fraca), respectivamente, p < 0,050.

### Confiabilidade interavaliadores

Houve diferença significante na confiabilidade interavaliadores, sendo maior no GCR do que no GSR, média de 0,276 e 0,247, respectivamente, p < 0,001. Para essa análise, utilizou-se o coeficiente kappa. Os dois grupos apresentaram concordância mínima.

### Correlação entre número de dimensões vocais e acurácia da análise

Os resultados da Tabela 1 mostram que a acurácia da análise dos alunos diferiu significantemente quanto ao número de dimensões vocais contido nos estímulos sonoros, em ambos os grupos e na amostra total, p < 0,001. A amostra de vozes contou com 17% de vozes com uma dimensão, 65,5% de vozes com duas dimensões e 17,5% de vozes com três dimensões.

**Tabela 1:** Valores descritivos e análise comparativa do desempenho na tarefa de identificação do desvio vocal predominante, de acordo com o número de dimensões vocais (1 - unidimensional, 2 – bidimensional, 3 – mais de duas dimensões) para o GSR, GCR e amostra total

**Variável**	**Grupos**	**Número de Dimensões vocais**	**Média**	**DP**	**Mediana**	**Mín.**	**Máx.**	**Valor de p**	**Post-hoc**	**Valor de p**	^r^
**Acurácia (%)**	**GSR**	1	72,29	14,65	76,47	26,47	91,18	**< 0,001**a*	1D x 2D	**0,001**	0,577
		2	50,70	6,46	49,62	37,40	64,89		1D x 3D	**0,001**	0,635
		3	48,46	9,61	51,43	31,43	65,71		2Dx 3D	0,999	0,058
	**GCR**	1	67,65	10,12	67,65	52,94	85,29	< 0,001^a^*	1D x 2D	**0,001**	0,500
		2	49,54	6,25	50,00	35,88	61,83		1D x 3D	**0,001**	0,946
		3	41,94	7,93	42,86	28,57	54,29		2Dx 3D	**0,003**	0,446
	**Total**	1	69,88	12,61	73,53	26,47	91,18	**< 0,001**b*	1D x 2D	**0,001**	1,569
		2	50,10	6,32	49,62	35,88	64,89		1D x 3D	**0,001**	1,967
		3	45,0	9,30	45,71	28,57	65,71		2Dx 3D	**0,001**	0,795

**Legenda:** D: dimensões; DP: Desvio padrão; Mín.: Mínimo; Máx.: Máximo

ANOVA de Friedman (^a^) e ANOVA com um fator de medidas repetidas (^b^)

Teste para tamanho de efeito: coeficiente r (^r^)

* valor estatisticamente significante p ≤ 0,05

O GCR apresentou maior porcentagem de acertos na classificação de vozes com uma dimensão alterada em relação a vozes com duas dimensões alteradas ou três dimensões alteradas. A porcentagem de acertos para vozes com duas dimensões alteradas apresentou diferença significante em comparação à de três dimensões alteradas, com maior porcentagem de acertos para as vozes com duas dimensões alteradas. A análise *post hoc*, realizada com o teste de postos sinalizados de Wilcoxon com correção de Bonferroni, foi utilizada nestas análises.

Para vozes com uma dimensão alterada, os estudantes do GSR apresentaram diferença significante na porcentagem de acertos em relação a vozes com duas e três dimensões alteradas, sendo que as vozes com uma dimensão alterada tiveram maior porcentagem de acertos. A porcentagem de acertos nas vozes com duas e três dimensões alteradas foi semelhante.

Para a amostra total, a análise *post hoc*, realizada com o teste de Bonferroni, mostrou que, ao classificar vozes com uma dimensão alterada, houve diferença significante na porcentagem de acertos em relação à condição de duas ou três dimensões alteradas, com maior porcentagem de acertos para vozes com uma dimensão alterada. Além disso, houve maior porcentagem de acertos para a condição de duas dimensões alteradas do que com três dimensões. Sendo assim, quanto maior o número de dimensões vocais no estímulo sonoro, menor a acurácia dos participantes. A Figura 2 apresenta a média de acertos para a tarefa de identificação de vozes de acordo com o número de dimensões alteradas e o grupo.

**Figura 2:** Média de acertos para a tarefa de identificação de vozes de acordo com o número de dimensões alteradas e o grupo

### Número de dimensões vocais: análises de regressão linear múltiplas

Os modelos de regressão linear múltipla foram elaborados para verificação da capacidade preditora do número de dimensões vocais em relação ao desempenho na tarefa de identificação do desvio vocal predominante, para cada grupo e amostra total. Nestes modelos, a porcentagem de acertos para cada estímulo sonoro foi considerada variável dependente e o número de dimensões alteradas como variável independente. O tipo de dimensão na voz - rugosa, soprosa ou tensa - foi inserida como primeiro passo nos modelos, de modo a controlar seu efeito. A variável independente, ou seja, o número de dimensões vocais alteradas, foi inserida no segundo passo.

Os resultados mostraram melhora significativa no modelo de regressão linear em relação à capacidade preditora do desempenho na tarefa de identificação do desvio vocal predominante após a inserção do número de dimensões alteradas, ordem indireta. A variância do número de dimensões alteradas foi capaz de explicar 12%, 16,1% e 16% da variância observada no GSR, GCR e amostra total, respectivamente, no desempenho da tarefa de classificação do desvio predominante. A variável número de dimensões vocais contribuiu de forma significante para os modelos, p < 0,001, sendo que o coeficiente não-padronizado b sugeriu que, controlando as demais variáveis do modelo, o aumento de uma dimensão alterada leva à redução de 13,13% de acertos no GSR, 15,51% de acertos no GCR e 14,36% de acertos na amostra total do estudo. Sendo assim, o número de dimensões vocais foi um preditor significativo no desempenho da tarefa de identificação dos desvios vocais predominantes, para ambos os grupos e para amostra total, Tabela 2.

**Tabela 2:** Modelo de regressão linear múltipla do número de dimensões alteradas como preditora do desempenho na tarefa de identificação de vozes para o GSR, GCR e para amostra total

**Passo**		**b**	**β**	**Valor de p**
**Grupo Sem Repetição de Âncoras – GSR**				
1	Constante	58,28	--	**< 0,001** *
	Tipo de alteração predominante	-2,23	-0,083	0,240
		-5,81, 1,34		
2	Constante	88,61	--	< 0,001*
	Tipo de alteração predominante	-4,31	-0,161	0,019*
		-7,49, -0,96		
	Número de dimensões alteradas	-13,13	-0,355	< 0,001*
		-18,11, -8,03		
**Grupo Com Repetição de âncoras – GCR**				
1	Constante	63,72	--	< 0,001*
		56,04, 71,18		
	Tipo de alteração predominante	-6,46	-0,209	0,003*
		-10,75, -1,91		
2	Constante	99,56	--	< 0,001*
		85,52, 113,32		
	Tipo de alteração predominante	-8,92	-0,289	< 0,001*
		-12,54, -4,72		
	Número de dimensões alteradas	-15,51	-0,363	< 0,001*
		-21,33, -9,73		
**Amostra Total**				
1	Constante	61,10	--	< 0,001*
	Tipo de alteração predominante	-4,42	-0,164	0,020*
		-8,32, -0,67		
2	Constante	94,28	--	< 0,001*
		81,88, 105,73		
	Tipo de alteração predominante	-6,70	-0,249	< 0,001*
		-10,03, -3,34		
	Número de dimensões alteradas	-14,36	-0,386	< 0,001*
		-19,14, -9,66		

r^2^ = 0,007 (p = 0,240) para Passo 1; r^2^ = 0,120 (p < 0,001*) para Passo 2 para GSR; r^2^ = 0,044 (p = 0,003*) para Passo 1; r^2^ = 0,161 (p < 0,001*) para Passo 2 para GCR; r^2^ = 0,027 (p = 0,020*) para Passo 1; r^2^ = 0,160 (p < 0,001*) para Passo 2 para amostra total

* valor estatisticamente significante p ≤ 0,05

### Relação entre o desvio vocal predominante e a acurácia da análise

A comparação da análise do GCR e do GSR com a de referência mostrou que houve diferença significante entre os grupos na acurácia das vozes tensas. O GSR apresentou maior acurácia na classificação de vozes tensas do que o GCR, p<0,001. Nos demais desvios vocais, os grupos tiveram desempenhos semelhantes, Tabela 3.

**Tabela 3:** Valores descritivos e análise comparativa dos grupos em relação às porcentagens de acertos e de respostas sem predomínio na tarefa de classificação do desvio vocal predominante

**ACURÁCIA**	**Grupo**	**Média**	**DP**	**Mediana**	**Mín.**	**Máx.**	**Valor de p**	**T.E.**
**Voz Rugosa**	GSR	55,86	9,32	54,88	41,46	71,95	0,992^a^	0,003^d^
	GCR	55,84	10,27	56,10	26,83	73,17		
**Voz Soprosa**	GSR	57,13	10,04	59,34	32,00	70,67	0,556^a^	0,148^d^
	GCR	58,62	8,40	58,00	45,33	73,33		
**Voz Tensa**	GSR	50,12	9,80	50,79	30,16	66,67	**< 0,00**1*a	1,019d
	GCR	40,14	9,04	39,68	25,40	55,56		
**Sem predomínio**	GSR	2,15	3,28	0,45	0,00	10,00	0,121b	0,220r
	GCR	3,07	3,64	1,82	0,00	13,18		

**Legenda:** DP: Desvio padrão; Mín.: Mínimo; Máx.: Máximo; T.E.: Tamanho do efeito

Teste t de Student para amostras independentes (^a^) e Teste U de Mann-Whitney (^b^)

* valor estatisticamente significante p ≤ 0,05

Testes para tamanho de efeito: coeficiente r (^r^) e coeficiente d (^d^)

A análise *post hoc*, realizada com o teste de Bonferroni, revelou que ao considerar todas as vozes avaliadas pelos estudantes, a repetição de estímulos âncoras não se associou a uma maior ou menor acurácia, não demonstrando efeito significante do grupo, p = 0,072, r = 0,247. Entretanto, para o desvio vocal predominante, houve efeito significante, p < 0,001, r = 0,600. Observou-se diferença entre “tensão” e “rugosidade”, p < 0,001, e entre “tensão” e “soprosidade”, p < 0,001, sendo que a tensão teve a menor acurácia. Não houve diferença entre “rugosidade” e “soprosidade”, p = 0,743. Portanto, considerando os dois grupos, vozes predominantemente tensas apresentaram menor acurácia em comparação às vozes com predomínio de rugosidade ou de soprosidade, que foram semelhantes entre si. A acurácia da análise relacionada ao tipo de dimensão vocal diferiu de acordo com o grupo, com interação estatisticamente significante, p = 0,003, r = 0,323.

Para verificação da ocorrência de alta ou baixa acurácia para as vozes soprosas, rugosas ou tensas, os estímulos sonoros foram organizados em dois grupos: ocorrência de acertos ≥ a 75% e < 75%. Ao relacionar a taxa de acertos com a amostra total de vozes, os resultados apontaram que as proporções de vozes soprosas, rugosas e tensas foram semelhantes nos grupos de ocorrência de acertos maior ou igual 75% e menor que 75%, tanto para GSR, quanto para GCR. No entanto, ao considerarmos a amostra total do estudo, observou-se que a voz soprosa predominou na distribuição de vozes com acurácia ≥ 75%, Tabela 4.

**Tabela 4:** Comparação dos tipos de vozes em relação à acurácia para cada grupo e para a amostra total

**GRUPO**	**Desvio Vocal Predominante**	**Acurácia da Análise**			**Valor de p**
		**Alta**	**Baixa**	**Total**	
		Número (%)	Número (%)	Número (%)	
**GSR**	R	12 (28,57)	62 (39,24)	74 (37,00)	0,311
	S	18 (42,86)	49 (31,01)	67 (33,50)	
	T	12 (28,57)	47 (29,75)	59 (29,50)	
**GCR**	R	11 (25,58)	63 (40,13)	74 (37,00)	0,053
	S	21 (48,84)	46 (29,30)	67 (33,50)	
	T	11 (25,58)	48 (30,57)	59 (29,50)	
**Total**	R	9 (25,71)a	65 (39,39)^a^	74 (37,00)	**0,023** ^*^
	S	19 (54,29)b	48 (29,09)^a^	67 (33,50)	
	T	7 (20,00)^a^	52 (31,52)^a^	59 (29,50)	

As letras (^a,b^) indicam subconjuntos da variável “Grupo” cujas proporções das colunas não se diferem significativamente umas das outras no nível de significância de 5% (p ≤ 0,05).

* valor estatisticamente significante p ≤ 0,05

### Relação entre a acurácia e a fadiga autorreferida pelos participantes

Foi realizada a análise de variância univariada (ANOVA) de efeitos mistos para comparar o efeito da fadiga autorreferida pelos estudantes com a acurácia de suas respostas. Para esta análise, foram considerados apenas 23 participantes do GSR e 21 participantes do GCR, pois alguns participantes destes grupos não marcaram cansaço durante a tarefa de classificação de vozes. A porcentagem média da acurácia no momento pré-fadiga para o GSR foi de 55,77% e para o GCR foi de 55,18%. Já no momento pós- fadiga, o GSR apresentou uma porcentagem média 53,91% e o GCR de 52,65%. O tamanho do efeito dos fatores foi medido por meio do cálculo do coeficiente r, calculado a partir da conversão da estatística F proposta por Field. Não foram observadas interações estatisticamente significantes entre os fatores “Grupo” e “Momento Fadiga” para a porcentagem de acertos, p = 0,788.

Sendo assim, a diferença entre os participantes dos grupos com e sem repetição de âncoras, em relação à acurácia na tarefa de classificação de vozes, não foi acentuada pela fadiga autorreferida, tornando os momentos pré e pós-fadiga semelhantes entre si.

## DISCUSSÃO

A avaliação perceptivo-auditiva da voz tem grande valor clínico e por isso é amplamente utilizada na rotina de atendimento^(5)^. Entretanto, por ser uma análise subjetiva, pode ser susceptível a erros sistemáticos e aleatórios^(12)^. Erros sistemáticos na APA são mais passíveis de controle, mas torna-se necessário conhecer suas fontes de erros. Um dos fatores que influenciam a APA é o treinamento perceptivo-auditivo dos juízes. Avaliadores inexperientes necessitam de pelo menos 8 horas de treinamento para obter confiabilidade aceitável^(21)^. O treinamento perceptivo, seja com vozes humanas ou sintetizadas, torna possível a criação de referências internas dos estímulos sonoros. Outro importante fator de interferência na APA é o uso de estímulos âncoras, que possibilita a calibração da percepção auditiva dos avaliadores.

A tarefa de fala escolhida também influencia a APA. No presente estudo, a tarefa de fala utilizada foi a emissão sustentada da vogal /ε/ em frequência e intensidade habituais. A tarefa de vogal sustentada é frequentemente utilizada na prática clínica e em pesquisas científicas por oferecer informações sobre as características vocais com foco na fonte glótica, sem interferências dos aspectos articulatórios e de prosódia^(7)^. Desvios vocais como rugosidade e soprosidade são produzidos pela laringe, que funciona como transdutor da energia aerodinâmica, originada pelo fluxo de ar vindo dos pulmões, em energia acústica^(22)^. A tensão pode ser produzida na laringe e ao longo do trato vocal. Dessa forma, o uso de vogal sustentada no treinamento perceptivo-auditivo de vozes torna-se uma tarefa importante na identificação dos desvios vocais predominantes.

Os parâmetros internos dos avaliadores inexperientes são relativamente estáveis para julgar vozes normais ou próximas da normalidade. Isto se deve ao contato e experiência com vozes normais, que servem de base para seus julgamentos perceptivos^(2)^. Para que treinamentos perceptivo-auditivos possam ser planejados de modo mais eficiente, é fundamental que a percepção auditiva de base dos alunos em formação seja conhecida. Os participantes de ambos os grupos tiveram desempenhos semelhantes na acurácia da análise do desvio vocal predominante, sendo de 54,65% no GSR e de 52,29% no GCR. De certa forma, a acurácia baixa já era esperada, uma vez que os participantes eram alunos de graduação, ainda com pouca experiência na avaliação de vozes disfônicas. Numa pesquisa realizada com fonoaudiólogos de um curso de especialização em voz, o valor médio da acurácia de acerto na classificação de vozes predominantes foi de 72%^(23)^.

A repetição de estímulos âncoras ao longo da avaliação perceptiva não aumentou a acurácia da análise dos alunos do GCR, sendo esse resultado contrário à hipótese inicial do estudo. Repetir um estímulo coopera para que cérebro reconheça os padrões de forma mais rápida e contribui para o refinamento da percepção das características do estímulo apresentado^(20)^. Embora a acurácia não tenha aumentado, a estratégia de repetir os âncoras resultou em maior confiabilidade intra e interavaliadores no GCR, mas ainda com concordâncias baixas. Esta diferença entre os grupos sugere que o GCR pôde fazer ajustes em seus padrões internos^(16,24,25)^.

Julgar a qualidade vocal de um estímulo sonoro da mesma forma em momentos diferentes configura concordância perceptivo-auditiva. A concordância é um desafio até mesmo entre juízes experientes, pois é influenciada por diversos fatores^(6,18)^. Como visto neste estudo, o uso de referências auditivas pôde contribuir de forma discreta para o aumento da concordância entre os participantes. De acordo com a literatura^(16,24,26,27)^, o treinamento perceptivo-auditivo com âncoras em ouvintes inexperientes na classificação de vozes desviadas é eficaz no aumento da acurácia das classificações e que ouvintes sem experiência podem apresentar o mesmo grau de concordância intra e interavaliadores que o ouvintes experientes após o treino. Dessa forma, o uso de estímulos âncoras tem mostrado grande potencial na redução da variabilidade das classificações das vozes e no aumento da concordância intra e inter-avaliador de ouvintes inexperientes, compactuando com o pressuposto inicialmente. O aumento da confiabilidade aponta que os participantes puderam se ajustar às referências externas, consolidando e ajustando seus padrões internos^(25)^. Contudo, ainda que o GCR tenha apresentado maior confiabilidade que o GSR, a concordância foi mínima. Isto sugere a necessidade de treinamentos perceptivo-auditivos constantes ao longo da formação dos alunos de graduação.

Com relação à confiabilidade interavaliadores, avaliadores inexperientes apresentaram melhora na confiabilidade com apenas duas horas de treinamento com estímulos âncoras de diferentes tipos de desvios vocais^(16)^. Quando inseridas no contexto de avaliação e classificação de vozes, os estímulos âncoras diminuem ou até mesmo eliminam a dependência dos variados padrões internos, atenuando interferências e aumentando a concordância entre os avaliadores experientes e inexperientes^(18,24)^. Neste estudo, a concordância interavaliadores foi mínima em ambos os grupos, menor que a confiabilidade intra-avaliadores, mas significantemente maior no GCR. Dessa forma, o uso de estímulos âncoras para calibração da percepção auditiva dos alunos pode ser um recurso positivo na avaliação de vozes desviadas.

Vozes humanas desviadas podem conter um único tipo de desvio vocal, como as vozes soprosas de alguns casos de paralisias de prega vocal, ou um sinal mais complexo, com duas ou mais dimensões vocais associadas. A avaliação de estímulos sonoros mais complexos exige do ouvinte maior refinamento perceptivo-auditivo, o que dificulta a identificação do desvio vocal predominante^(1,16)^. Nessa pesquisa, o aumento do número de dimensões vocais contido nos estímulos sonoros, associou-se a uma diminuição na acurácia dos avaliadores, Tabela 1 e Figura 2, achados estes, que acordam com nossa hipótese inicial.

Sabendo que há fatores que podem interferir na classificação de vozes disfônicas^(12)^, o aumento do número de dimensões vocais nos estímulos sonoros foi um preditor significativo na classificação do desvio vocal predominante. Embora os valores tenham sido de 16,1% no GCR e de 12% no GSR, o número de dimensões vocais contidos nos estímulos respondeu por parte dos possíveis erros sistemáticos da APA, Tabela 2. Assim, o controle do número de dimensões vocais contido nos estímulos sonoros deve ser considerado no planejamento do treinamento auditivo de ouvintes inexperientes, iniciando-se com estímulos mais simples e evoluindo com estímulos mais complexos^(1)^.

Apesar da acurácia dos GSR e GCR ter sido semelhante na classificação das vozes soprosas e rugosas, o GSR apresentou maior porcentagem de acertos de vozes tensas quando comparado ao GCR, Tabela 3, mesmo sem âncoras auditivas repetidas ao longo da sessão de escuta. Dessa forma, a repetição de âncoras não contribuiu para a melhora da referência interna do parâmetro tensão, visto que o GCR não se destacou nesta análise quando comparado ao GSR^(6,11,16)^. De acordo com a literatura, o parâmetro tensão apresenta maior dificuldade de análise e menor confiabilidade^(1,12,17)^.

Ouvintes podem ter maior sensibilidade a qualidades vocais específicas pelo fato de o estímulo ser mais facilmente reconhecido e identificado. A voz soprosa se destacou por ser o tipo de desvio vocal de maior acurácia, Tabela 4. Na análise da amostra total de participantes, observou-se maior acurácia na classificação das vozes soprosa e menor na de vozes tensas, como já era esperado. As qualidades vocais tensa e rugosa não tiveram alta taxa de acertos (≥ 75%) como a voz soprosa. De fato, as vozes desviadas podem ser instáveis e apresentar mais de um parâmetro qualitativo^(16)^ e determinadas dimensões vocais podem ser mais facilmente percebidas e mentalmente armazenadas do que outras.

O grau de experiência de avaliadores que classificam vozes pode interferir na APA, uma vez que indivíduos inexperientes não tiveram exposição a vozes desviadas e, portanto, não formaram padrões internos precisos quanto aos tipos de desvios vocais. A experiência implica em alocar os padrões internos de forma consistente ao longo da avaliação dos desvios vocais e discernir as dimensões em questão^(16)^.

A amostra de estímulos sonoros utilizada neste estudo foi extensa, 220 vozes. A duração da APA do GCR foi muito maior que a do GSR. Embora a fadiga possa reduzir o desempenho cognitivo após longos períodos de atividade que demande atenção^(28-30)^, neste estudo, a fadiga autorreferida pelos estudantes não interferiu diretamente nos valores de acurácia das classificações, tanto no momento inicial das classificações, quanto no momento final.

A fim de refinar a percepção auditiva, treinamentos auditivos graduais a partir dos primeiros anos letivos podem contribuir para que o grau de concordância e de acurácia entre graduandos aumentem^(16,24)^. A partir dos resultados da pesquisa, pode-se sugerir que o treinamento perceptivo-auditivo de alunos da graduação utilize estímulos âncoras e que seja iniciado com estímulos sonoros mais simples, unidimensionais, evoluindo para os mais complexos; e com dimensões vocais mais concordantes, como a soprosidade e rugosidade, progredindo para dimensões menos concordantes, como a tensão.

Vozes desviadas frequentemente apresentam mais de um tipo de desvio vocal na APA, o que dificulta a avaliação principalmente por indivíduos inexperientes. Os estímulos âncoras têm se revelado uma ferramenta clínica útil no treinamento de ouvintes com dificuldade em manter a confiabilidade intra e intervaliadores na classificação de vozes^(15,16)^. Entretanto, as características da amostra vocal também devem ser consideradas na elaboração do treinamento perceptivo-auditivo, como a complexidade dos estímulos sonoros e os tipos de desvios vocais.

## CONCLUSÃO

A repetição de estímulos âncoras não aumentou a acurácia de classificação do desvio vocal predominante. Houve aumento da confiabilidade intra e interavaliadores no GCR, mas ainda se mostrou baixa. A complexidade dos estímulos sonoros interferiu na acurácia de ambos os grupos. Quanto mais simples o estímulo, maior a acurácia na identificação do desvio vocal predominante. O número de dimensões vocais foi um preditor na classificação do desvio. O tipo de desvio vocal também interferiu na acurácia, sendo que as vozes soprosas tiveram maior taxa de acertos.
